# Agrammatic aphasia verb and argument patterns in Kiswahili-English spontaneous language

**DOI:** 10.4102/sajcd.v62i1.89

**Published:** 2015-06-08

**Authors:** Hillary K. Sang

**Affiliations:** 1 Department of Linguistics, Moi University, Kenya

## Abstract

**Background:** The spontaneous and narrative language of Kiswahili agrammatic aphasic and non-brain-damaged speakers was analysed. The bilingual participants were also tested in English to enable comparisons of verb production in the two languages. The significance of this study was to characterise bilingual Kiswahili-English spontaneous agrammatic output. This was done by describing Kiswahili-English bilingual output data with a specific focus on the production of verbs. The description involves comparison of verb and argument production in Kiswahili and English.

**Methods and procedures:** The participants recruited for this study were drawn from two groups of participants (six non-fluent aphasic/agrammatic speakers and six non-brain-damaged). From each participant, a sample of spontaneous output was tape-recorded in English and Kiswahili based on the description and narration of the Flood rescue picture’ and the ‘Cookie theft picture’. The data elicited were compared for each subject and between the participants and relevant verb parameters have been analysed. The variables that were studied included mean length of utterance (MLU), inflectional errors, verb tokens and types, copulas and auxiliaries. Further, all verbs produced were classified as per their argument structure.

**Results:** The results from English data supported previous findings on agrammatic output. The agrammatic participants produced utterances with shorter MLU and simpler sentence structure. However, Kiswahili data surprisingly showed reversed results, with agrammatic speakers producing longer utterances than non-brain-damaged (NBD) controls. The results also revealed selective impairment in some agrammatic speakers who made inflectional errors. The verb argument structure showed contrasting results, with agrammatic speakers preferring transitive verbs whilst the NBD speakers used more intransitive verbs.

**Conclusions:** The study attempts for the first time to characterise English-Kiswahili bilingual spontaneous and narrative output. A quantitative analysis of verb and argument production is conducted. The results of the English data are consistent with those in the literature; agrammatic speakers produce utterances with shorter MLU and simpler sentence structure. However, Kiswahili data reveals a surprisingly reversed pattern most notably with respect to MLU with agrammatics producing longer utterances than NBD controls. Argument structure analysis revealed that agrammatics used more transitive verbs than intransitives.

## Introduction

Aphasia is caused by a focal brain injury after language acquisition due to a stroke, traumatic brain injury or tumour. This may lead to agrammatism, traditionally defined as a disorder of language production that is a clinical syndrome of Broca's aphasia. Globally, the World Health Organization (WHO) estimated as far back as 2004 that 15 million people worldwide suffered from a stroke annually. Out of these, 5 million die, whilst another 5 million become permanently disabled. The burden of care is placed on families and communities. Africa accounts for 8% of all first-ever strokes and an estimated 5% of stroke survivors worldwide live in Africa according to WHO. Feigin, Lawes, Bennett, Barker-Collo and Parag (2009) predict that the prevalence of stroke might rise in future due to increased exposure to risk factors such as sedentary lifestyles. In Kenya, the Centre for Disease Control (CDC) lists stroke among the top ten causes of death in the country. This study takes a linguistic perspective on the manifestation of aphasia due to stroke. Kiswahili-English language therapists need this kind of information to correctly assess and determine whether their patients present with agrammatism (CDC-Kenya 2010).

Agrammatism is an acquired language disorder resulting from left hemisphere brain damage, which is characterised by simplification of structure and the omission and/or substitution of inflectional morphemes (e.g. Goodglass, 1968; Marshall, 1986). Studies on agrammatic output in the literature (for example, Thompson, Shapiro, Li & Schendel, 1994; Vermeulen, Bastiaanse & Van Zonneveld, 1998) typically compare production of nouns and verbs showing that the former are less problematic for agrammatic individuals. However, there are very few studies of this kind on other languages.

Several other studies looking specifically at the verb (e.g., Bastiaanse, Jonkers & Moltmaker-Osinga, 1996; Saffran, Berndt & Schwartz, 1989; Thompson, Shapiro, Li & Schendel, 1994; Thompson, Lange, Schneider & Shapiro 1997) have found that agrammatic speakers are impaired in verb production. The production of verbs and their diversity are significantly reduced, whilst verb inflections and auxiliaries are often omitted. Grodzinsky (1984) observed that the pattern of omission or substitution is morphologically driven and depends on whether the stem can be a free-standing morpheme or not.

Rossi & Bastiaanse (2008) investigated verb production in a group of Italian agrammatic speakers. They found that agrammatic speakers are impaired in verb production with omission of verbs and inflectional errors characterising their output. In comparison to non-brain-damaged speakers, their agrammatic speakers produced fewer lexical verbs, fewer modal verbs and fewer auxiliaries. However, they found that copula production was similar in the two groups. In conclusion, they hypothesised that agrammatic speakers have a deficit in grammatical encoding as shown by their patterns of omissions and inflectional mistakes of verbs. Also, agrammatic speakers overuse verbs without internal arguments, whereas the proportion of verbs with internal arguments is reduced compared to output of healthy people (Thompson, 2003).

Recent studies by Abuom and colleagues on Kiswahili-English agrammatism have shown a distinct selective deficit for production and comprehension of verb forms (Abuom & Bastiaanse, 2013; Abuom, Obler & Bastiaanse, 2011). The same finding is reported in their study that analysed spontaneous output of agrammatic aphasic. They found that the impairment was more severe in English despite its simpler verb paradigm than Kiswahili. Their results further demonstrated that Kiswahili-English agrammatic aphasics had difficulty comprehending passive sentences. This study builds on these findings by looking at features of the verb as well as the argument structure of Kiswahili-English agrammatic speakers’ language.

The main focus in this study, like in Rossi & Bastiaanse (2008) was on verb and argument production, but unlike their monolingual Italian speakers, the present study analysed the spontaneous and narrative output of the English-Kiswahili bilinguals, both agrammatic and age and education-matched non-brain-damaged speakers. Previous studies have unanimously found that verbs produced by agrammatic speakers are simpler in argument structure in comparison to those produced by non-brain-damaged speakers. The current study hypothesises that the same results will be found, at least in English.

The following parameters of the verb paradigm were analysed:

1. verb omissions and inflection
2. verb tokens and types
3. use of copulas, modals, and auxiliaries
4. verb argument structure.

The comparison of bilingual agrammatic and non-brain-damaged speakers of English and Kiswahili was based on the parameters outlined above. Similarities and differences between the two groups are of particular interest. This was important in the determination of the severity of verb production problems in the agrammatic group. The structural and morphological differences between the two languages imply that differences were likely to be observed. The next section discusses the language situation in Kenya, followed by a description of the relevant aspect of the Kiswahili language and its grammar. Subsequently, some relevant issues with respect to bilingualism and aphasia will be explored briefly. Finally, the purpose, aims and research questions for the present study will be presented.

### Sociolinguistic situation in Kenya

Kenya is a multilingual country in which over 40 languages are spoken. However, English and Kiswahili dominate in that they are given official recognition, whilst other indigenous languages are not. English is used in education, for official purposes and international communication, whilst Kiswahili is the national language and is used in the political arena, parliament, and as a language of political unity and national identity (Kembo-Sure, Ogechi & Mwangi, 2006).

This study investigates agrammatic and non-brain-damaged speakers of Kiswahili and English to find out the differences in verb production in these two languages with strikingly different verb morphology. In the Kenyan context, the languages being studied here are acquired at the same time (kindergarten level), effectively making the speakers bilingual. However, strictly speaking, the participants are trilingual given that their native languages all belong to one of the more than 40 ethnic languages spoken in Kenya. It is only Kiswahili and English that are taught as school subjects from kindergarten to university, therefore proficiency levels can be assumed to be at par.

### Brief description of Kiswahili

Whiteley (1974) classifies Kiswahili as belonging to the large family of Bantu languages and as native to the peoples who live on the East coast of Africa that stretches from the south of Somalia to the north of Mozambique, including the islands of Pate, Lamu, Pemba, Zanzibar and Mafia. From east to west, the area of influence of Kiswahili extends from Tanzania and Kenya through the interior of Congo up to Uganda, Burundi, Zambia and Malawi.

The present study attempts to characterise the production of verbs in English-Kiswahili speakers. These languages are very different with respect to their verb inflection paradigms. However, there are some similarities between Kiswahili and English, mainly in sentence structure configuration; both have a basic subject-verb-object (SVO) configuration (for example, ‘the boy (S) kicked (V) the ball (O)’). Both languages also allow inverted constructions such as passives, wh-questions, relative and object clefts that change the argument structure of the verb.

The main characteristic of the Kiswahili verb that differentiates it from its English counterpart is its agglutinative aspect. Agglutinative languages are those that have affixes representing various grammatical markers glued to the verb root (see example 2 in section 1.3.1 below). According to Omondi (1999), the verb becomes a functional part of the sentence, when a certain number of affixes are attached to it: prefixes, infixes and suffixes, according to the situation. All these affixes possess a precise position and function. The general position schema of these affixes in relation to the verb radical is as follows:

#### Verb paradigm

1. Pre-prefix + subject prefix + tense marker + object infix + ROOT + derivation + suffix + post-suffix
  There are however very few verbs which contain this full representation of the verb paradigm.
*Example:*
2. A –   li– m–  pig-a
  Subject prefix PST   OBJ ROOT + final vowel/derivation (VERB STEM)
  (I)      (him) (beat)

The verbal morphology of Kiswahili is clearly more complex than that of English, involving numerous inflectional and derivational morphemes. Verbal prefixes are associated with inflection: the main ones are subject and object agreement markers, and tense (relative clauses and reflexives are expressed by means of special object markers). The verbal suffixes are derivational morphemes. The most frequent are the causative, passive, stative, applicative, and reciprocal. The subject and object markers agree in gender and number with the appropriate argument. Subject agreement is almost always mandatory for finite verbs, but the use of the object marker is optional. Object marking is possible with every semantic class of objects, although it is more frequent with animate objects.

#### Copula and auxiliary verbs

Universally, copulas are not regarded as verbs in the strict sense but as lexicalisations of inflection. Unlike lexical verbs, copulas and auxiliaries in general are indeed assumed to be base-generated (projecting from the inflectional phrase- IP) rather than being generated in the verb node. Copulas in Kiswahili have little independent meaning and mainly function to relate sentential elements of clause structure, especially subject and complement. Examples of copula verbs are; NI, SI, NDI-, -LI-, -PO, -KO,-MO,-NA,YU, U and WA. (Ashton, 1982).

In Kiswahili, Auxiliary verbs accompany main verbs to express a special aspect of an action, for example:

3. A - li- kuwa  a- na- kunywa chai
  Subject prefix PST COP subject prefix PresT STEM   OBJ
  (He   was    drinking tea)
4. Kijana  a- ta-  taka kuenda nyumbani
  Subject (subject prefix)  FutT COP INF  LOC
  (The boy       will  Want to go home)

In example 3, the –li- marker in the auxiliary *kuwa* (to be) indicates that the activity started in the past, whilst in example 4, the –ta- marker in the auxiliary *taka* indicates that the activity will take place in future.

Auxiliary verbs are used to make distinctions in relation to mood, aspect, voice and so on. Examples of auxiliary verbs are: kuwa (be), weza (can), pata (get) and wahi (be in time).

### Aphasiological perspective

Fabbro (2001) observes that grammatical deficits in aphasia depend on the structure of the language system. This means that problems with verb production and argument structure faced by agrammatic speakers are a reflection of the complexity (or lack of it) in the morphosyntactic structures of their languages. Although English and Kiswahili have the same sentence configuration (SVO), their verbal paradigms as discussed earlier, differ significantly.

In the context of this study, the factor of bilingualism is also important in describing the aphasiological manifestations of the agrammatic speakers investigated. There is considerable literature on bilingual aphasia (see Albert & Obler, 1978; Fabbro, 1999; Paradis, 1995), and a growing number of published work in Kiswahili and other Bantu language family. One such study, Abuom et al. (2011) tests explanations of agrammatism using Kiswahili and English. They investigated the patterns and degree of severity of time reference impairments in bilingual agrammatic speakers of Kiswahili and English. However, time reference is not a concern for the present study.

The studies on bilingual aphasia have shown that bilingual aphasic speakers do not necessarily manifest the same language disorders with the same degree of severity in both languages. According to Paradis (1995), bilingual aphasic speakers should be assessed not only in one of their languages, but in both. Hence, this study tests bilingual agrammatic speakers in English and Kiswahili languages to determine the nature of impairment with respect to verb production.

### Theoretical perspective

Thompson et al. (1994) in their description of spontaneous output from agrammatic speakers reported the production of fewer verbs than non-brain-damaged speakers. They also found that agrammatic speakers tended to produce verbs with no internal arguments or, if any, just one internal argument. This, together with experimental results, led to the formulation of the Argument Structure Hypothesis (ASCH) (Thompson, 2003), which proposes that the increased complexity of verbal argument structure precludes more difficulty for agrammatic speakers. The current study describes verb production in Kiswahili, which has a more complex way of expressing argument structure than English. We hypothesise that at least English data will show results similar to those found by previous studies (e.g. Thompson, 2003). A comparison of the languages is expected to further affirm the ASCH, with agrammatic speakers showing more difficulty in Kiswahili than English.

### Aims

The main aim of the study is to describe Kiswahili-English bilingual output data with a specific focus on the production of verbs. The description therefore involved comparison of verb production in Kiswahili as well as in English.

### Purpose

The specific motivation for the study is:

1. To describe Kiswahili spontaneous and narrative output data with respect to verb production.
2. To compare the data with the analysis of English spontaneous and narrative output.

### Research questions

In characterising verb and argument production in Kiswahili-English bilingual agrammatics, the following issues are addressed:

1. Are Kiswahili agrammatic speakers impaired in verb and argument production?
2. Do they omit verbs in obligatory contexts?
3. Do they make inflectional errors?
4. Is the diversity of verbs produced limited?
5. Is production of lexical verbs, copulas and auxiliaries different from normal?
6. Do they prefer simpler or more complex argument structures compared to non-brain-damaged speakers?

### Method

#### Ethical considerations

The Graduate Research Ethics Committee of Moi University approved the study. In granting permission for the study, the ethics committee emphasised the need to maintain confidentiality and anonymity of participants and information collected from them. Although all participants could read and understand consent forms, they were read to the participants in the presence of an adult family member whom they selected as a witness. There was no need to translate consent forms, since all the participants were proficient in English and Kiswahili. All the participants understood what entailed their participation in the study and appended their signatures. To ensure confidentiality of participants, codes were used on the form and on interview transcripts instead of names. There was no disclosure whatsoever of the participants specific locations to avoid identification by unauthorised parties.

#### Participants

The participants recruited for this study were drawn from two groups of participants (six non-fluent aphasic/agrammatic speakers and six non-brain-damaged). They were matched on age and education level which was kept at a minimum of O-Level qualification. In the Kenyan school system, these are graduates who have gone through kindergarten, elementary (primary school) and high school tiers of the education system, which means 12 years of uninterrupted exposure to English and Kiswahili. All participants are right-handed and without any history of psychiatric or developmental output or language disorders or any other neurological conditions.

The agrammatic speakers produced telegraphic output in both languages confirmed by a practicing output therapist. Unfortunately, there are no tests available to establish the aphasia syndrome, but all agrammatic speakers had good comprehension in both languages on an adapted version of the subtask for auditory comprehension of single words (Boston Diagnostic Aphasia Examination [BDAE]-word comprehension test Goodglass & Kaplan, 1983). Their details are shown in the Table 1.

**TABLE 1:** Details of agrammatic speakers.

Participant	Age	Sex	Handedness	Education	Years post stroke	Classification	BDAE Results
SW	20	M	R	12	2	Non-fluent	71.5/72
HJ	45	F	R	12	10	Non-fluent	72/72
LA	43	F	R	16	1	Non-fluent	71.5/72
MM	46	F	R	16	16	Non-fluent	72/72
JK	49	M	R	17	1	Non-fluent	72/72
EA	40	M	R	16	17	Non-fluent	72/72

#### Materials

Participants were recruited through purposive sampling at Aga Khan University and Nairobi hospitals in collaboration with resident output and language therapists. The therapists were given a language profile of agrammatism and asked to recommend patients who exhibited the following characteristics: non-fluent language production, words produced limited to content words (nouns, verbs and adjectives), telegraphic output and articulation problems. From each participant, a sample of narrative output was tape-recorded in English and Kiswahili based on the description and narration of the ‘Cookie theft picture’ from the *Boston Diagnostic Aphasia Examination* (*BDAE*) (Goodglass & Kaplan, 1972) and the Pulitzer Prize winning photograph by Annie Wells, ‘Flood rescue picture’. These assessment tools were chosen, since Kenya does not have any test for identification of the aphasia syndrome. The elicitation method adopted was in the format of a semi-structured interview that involved the interviewer showing the participants the pictures and asking them, ‘Can you tell me what is happening in this picture?’

Narration involved participants being asked to tell a story from the pictures with ‘a beginning, middle and an end’ for both pictures. In both description and narration, participants were encouraged to tell as much as possible about the pictures.

A further tape-recording of spontaneous output was done to elicit the number of utterances required for analysis. Agrammatic participants were asked the following questions:

1. Can you tell me about your stroke?
2. Can you tell me about your work before the stroke?
3. Can you tell me about your family?
4. Can you tell me about your hobby?

For comparison purposes, questions 1 and 2 were slightly modified for the non-brain-damaged participants to ‘Can you tell me something about your last illness??’ and ‘Can you tell me about your past work?’ respectively.

#### Procedure

Previous studies have used varied sample sizes in their analyses of spontaneous output. Vermeulen et al. (1989) drew 300 words from the spontaneous output of their aphasic patients, whilst Berndt, Haendiges, Mitchum & Sandson (1997) did their analysis on samples of 150 words. Rossi & Bastiaanse (2008) used all spontaneous output materials elicited from every participant in their study. The present study ran analyses on samples of 200 words. However, since Kiswahili is an agglutinative language and English is not, comparisons on the basis of the number of words do not seem to be appropriate. For example, to express the past perfect in English, four words are need (e.g. ‘he had been writing’), whereas only two words are used in Kiswahili (‘amekuwa akiandika’). This study therefore based analyses on utterances extracted from the 200 word samples recorded from the participants. Utterances were defined as those clauses containing a verb meaning (i.e. verb, copula, modal or auxiliary) for purposes of comparison. Doing this, provided a similar number of utterances for both English and Kiswahili, thus enabling comparisons to be done statistically. So, from the 200 word samples in both English and Kiswahili, we had an average of 64 utterances. In essence the comparison between the two languages done in this study was based on the number of utterances and not the number of words.

The illustration below shows that whereas words are variable, utterances tend to be more stable as units of analysis. This is the reason why utterances as opposed to words were used for comparison between the two languages. The calculation of mean length of utterance (MLU) was done by dividing the number of words per utterance by the number of utterances.

1. Kiswahili: Alim**piga  ** - 1 word 1 utterance (MLU=1)
English: He **beat** him  - 3 words  1 utterance (MLU=3)
2. Kiswahili: Amekuwa a**kiandika** - 2 words  1 utterance (MLU=2)
English: He had been **writing** - 4 words 1 utterance (MLU=4)
NB: English has more words per utterance.

Recording sessions were held in a quiet setting for each of the participants using a digital audio recorder. The participants were asked to describe the pictures and then to tell a story, also based on the pictures, with a beginning, middle and an end. The samples collected were orthographically transcribed verbatim and then segmented into sentences. Hartmann and Stork's definition (1972) of a clause as a grammatical unit that includes, at minimum, a predicate and an explicit or implied subject and expresses a proposition, informed the criterion of segmentation. Thus, well-formed sentences with the elimination of repetitions and hesitation phenomena (e.g. eeeh, uum, well…) were of particular consideration.

#### Scoring

An utterance considered to be a unit of output bounded by breaths or pauses (Aronoff & Rees-Miller, 2001) was the unit of analysis critical in scoring. However, for the present study focus was on clauses that contained a verb, meaning that utterances containing verb, copula, modal, or auxiliary were analysed. The analyses were carried out on agrammatic and non-brain-damaged speakers’ samples with each lexical verb, auxiliary, copula, and modal verb being counted and scored. The finiteness of the verbs was also taken into account as well as inflectional errors. All these variables were counted and divided by the number of utterances for each speaker.

Further, the argument structure for each verb produced (following Rossi & Bastiaanse, 2008) was analysed for the number of internal arguments. In this respect, three verb argument structures were examined: intransitives, transitives and ditransitives. For all the analyses described here, comparisons were done within and between groups for both English and Kiswahili data. The data of the agrammatic speakers were compared to those of non-brain-damaged speakers for both languages.

#### Statistical analysis

Statistical analysis involved all the participants (agrammatic and non-brain-damaged speakers). The two sample groups were treated as being independent of each other in the obvious sense that they are separate samples coming from different sets of individual speakers. The individual measures in the agrammatic group are in no way linked with or related to any of the individual measures in the non-brain-damaged group, and vice versa. The version of the statistical t- test that was applied was therefore the one assuming ‘Unequal Sample Variances.’ The measures of dispersion used to describe results were the mean and standard deviation.

### Data analysis

The results reported here are for six agrammatic and six non-brained-damaged bilingual speakers of English and Kiswahili. All analyses were done on the basis of the utterance as the primary unit in which all grammatical elements are contained. This means that all variables were analysed in relation to the number of utterances for each subject. The implication is that the total number of a given grammatical element (e.g. copula or auxiliary) produced by a subject was divided by the total number of utterances produced by that subject. It was therefore possible to do direct comparisons within and between participants for English and Kiswahili on a proportional basis.

The following variables were counted and divided by the number of utterances for each subject:

1. Mean Length of Utterance (MLU): the mean number of words per utterance. It is predictable that Kiswahili, being agglutinative, yielded more words per utterance than English.
2. Inflectional errors: verb inflection omissions and substitution.
3. Verb tokens and types.
  a. Verb tokens: the total number of lexical verbs, copulas, modals and auxiliaries
  b. Verb types: the number of different verbs per sample of 200 words in order to compute lexical diversity.

4. Copulas and auxiliaries were counted.
5. Verb argument structure: Bastiaanse & Jonkers (1998) analysed 300 consecutive words from their participants’ samples. For the present study, however, 200 words were extracted and the number and nature of realised internal arguments scored. This was necessitated by the fact that some of the agrammatic speakers could not reach the 300-word threshold. There were three possible verb-argument structures considered: verbs without internal arguments (intransitives), verbs with one internal argument (transitives) and verbs with two internal arguments (ditransitives).

### Results

#### Mean length of utterance

Mean length of utterance was calculated for both agrammatic and non-brain-damaged speakers and a difference in MLU between English and Kiswahili computed for each group. The data for both groups showed variation in MLU, implying that both sets of participants produced longer utterances in English (M = 6.45, SD = 1.0) than in Kiswahili (M = 3.8, SD = 0.5) for NBD. This was predictable as illustrated earlier, given the different configurations of the languages. A t-test however revealed that the difference in MLU between the two languages was not significant for both groups.

For non-brain-damaged participants, English showed a higher level of MLU (M = 6.8, SD = 2.5) than Kiswahili (M = 3.8, SD = 1.6). This difference was also not significant (*t* (8) = 12.6, *p* > 0.05).

For agrammatics, English showed a higher level of MLU (M = 6.05, SD = 2.0) than Kiswahili (M = 4.3, SD = 1.6). This difference was also not significant (*t* (9) = 3.4, *p* > 0.05). The MLU of participant EA was the lowest in both languages (English = 4.2, Kiswahili = 3.1). His utterances consisted of short and simple sentences that fell far short of those produced by a control subject of his age, education and professional background. He scored 2.5 SD below the mean of the MLU of the control participants in English, whereas all the other agrammatic speakers fell within the normal range. In Kiswahili, however, agrammatic speakers had longer utterances (M = 4.3) than non-brain-damaged speakers (M = 3.8). This can be attributed to a tendency by the former group to use circumlocutions hence making their utterances longer.

#### Inflectional errors

The non-brain-damaged speakers do not make inflectional errors in either language. There were no omissions or substitutions of verbs in obligatory contexts and therefore no analysis of inflectional errors was done. The patterns observed in agrammatic speakers reveal some variation, but most of them show errorless performances comparable to their non-brain-damaged counterparts.

Some agrammatic speakers omitted inflectional endings in obligatory contexts. The worst performer in this respect was participant EA, whose majority of verbs lacked inflections of any kind. He simply produced the stem of the requisite verb in both English (69% error rate) and an even higher percentage in Kiswahili (92% error rate). Participants HJ (31%) and SW (3%) had fewer inflectional errors in English, whilst MM (12%) and SW (3%) had this error rate in Kiswahili. These results are shown in Table 4.

**TABLE 2:** Differences in mean length of utterance (MLU) values between English and Swahili.

Mean length of utterance analysis	English	Swahili
Non-brain-damaged speakers		
BK	6.4	3.8
DM	6.8	3.4
IA	6.2	4
JN	6.2	3.5
KM	7.1	4.2
NK	5.8	3.9
Mean values	6.45	3.8
Agrammatic speakers		
EA	4.2	3.1
HJ	5.7	3.4
JK	6.4	4.5
LA	7.7	4.3
MM	6.2	5.2
SW	6.1	5
Mean values	6.05	4.3

**TABLE 3:** Error analysis: agrammatic speakers.

Language	Subject	Number of errors	Number of utterances	%
English	EA	27	39	69
	HJ	9	29	31
	JK	-	-	-
	LA	-	-	-
	MM	-	-	-
	SW	1	31	3
Swahili	EA	23	25	92
	HJ	-	-	-
	JK	-	-	-
	LA	-	-	-
	MM	4	34	12
	SW	1	34	3

Percentage of errors produced in relation to number of utterances.

**TABLE 4:** Verb production.

Language	Subject	Lexical verbs		
		Tokens	Types	TTR
NBD speakers				
English	BK	43	28	0.66
	DM	49	33	0.67
	IA	46	28	0.61
	JN	45	27	0.6
	KM	51	36	0.7
	NK	46	27	0.57
	Mean values	46.67	29.83	0.64
Swahili	BK	57	31	0.54
	DM	66	30	0.46
	IA	51	29	0.57
	JN	79	34	0.43
	KM	57	29	0.51
	NK	64	30	0.47
	Mean values	62.3	30.5	0.51
Verb production: Agrammatic speakers				
English	EA	42	26	0.61
	HJ	34	18	0.57
	JK	30	20	0.67
	LA	32	24	0.75
	MM	35	18	0.51
	SW	28	17	0.6
	Mean values	33.5	20.5	0.62

Tokens and types in 200 words.

#### Verb types and tokens

The non-brained-damaged participants produced fewer verb tokens in English (M = 46.67) than Kiswahili (M = 62.3) in a sample of 200 words. The lexical verb types in the two languages are, however, similar in English (M =29.8) and Kiswahili (M = 30.5). A similar trend was found in the agrammatic group, although the margin of difference in means for this group was reduced: verb tokens in English (M = 33.5) and Kiswahili (M = 36.7); verb types in English (M = 20.5) and Kiswahili (M = 20.3). This implies that, whereas in Kiswahili more verbs (verb tokens) are produced, the diversity of verbs (verb types) is decreased. The type-token ration (TTR) was calculated by dividing the number of different verbs (the types) by the number of tokens giving a ratio (between 1.00 and 0.00) that indicated the rate of diversity: a high ratio means a great diversity, whilst a low ratio implies poor diversity and hence low lexical content (Vermeulen et al. 1989). The TTR values for English are higher for both groups (Non-brain-damaged = 0.64; Agrammatic = 0.62) than Kiswahili (Non-brain-damaged = 0.51; Agrammatic = 0.57) indicating that diversity is lower in Kiswahili spontaneous output. This is shown in Tables 5–6.

**TABLE 5:** Raw numbers and proportion of lexical verbs, copulas and auxiliary verbs in 200 words.

Language	Subject	Lexical verbs		Copulas (Number)	Auxiliary verbs (Number)
		Number	TTR values		
NBD speakers					
English	BK	43	0.66	16	22
	DM	49	0.67	19	19
	IA	46	0.61	20	16
	JN	45	0.6	15	21
	KM	51	0.7	21	16
	NK	46	0.57	18	17
	Mean values	46.7	0.64	18.2	18.5
Swahili	BK	57	0.54	27	16
	DM	66	0.46	21	24
	IA	51	0.57	31	29
	JN	79	0.43	23	29
	KM	57	0.51	20	30
	NK	64	0.47	29	18
	Mean values	62.3	30.5	25.2	24.3
Agrammatic speakers					
English	EA	42	0.61	0	1
	HJ	34	0.57	3	8
	JK	30	0.67	13	22
	LA	32	0.75	18	18
	MM	35	0.51	16	10
	SW	28	0.6	5	19
	Mean values	33.5	0.62	9.2	13
Swahili	EA	26	0.73	0	0
	HJ	53	0.58	5	5
	JK	39	0.54	8	11
	LA	36	0.61	15	5
	MM	34	0.44	4	4
	SW	32	0.44	11	12
	Mean values	36.7	0.57	7.2	6.2

**TABLE 6:** Verb argument structure.

Language	Subject	Transitive	Intransitive	Ditransitive
NBD speakers				
English	BK	46.3	61.7	2.1
	DM	51.3	72.9	1
	IA	43.9	58.5	4.8
	JN	52.8	59.7	3
	KM	35.5	68.1	1
	NK	41.7	56.9	2.3
	Mean values	45.3	63	2.4
Swahili	BK	23.3	66.5	3.4
	DM	30.2	65.1	1
	IA	15	61.7	8.3
	JN	17.5	67	3
	KM	22.1	60.8	5.6
	NK	27.7	57.9	1
	Mean values	22.6	63.2	3.7
Agrammatic speakers				
English	EA	33.3	30.7	2.6
	HJ	62.1	27.6	10.3
	JK	57.6	12.1	9.1
	LA	39.3	39.3	0
	MM	81.1	8.1	0
	SW	29	25.8	0
	Mean values	50.4	23.9	3.7
Swahili	EA	56	44	4
	HJ	54	40	6
	JK	60.5	23.7	13.2
	LA	48.6	27	8.1
	MM	61.3	29	3.2
	SW	52.9	26.5	0
	Mean values	55.6	31.7	5.8

### Lexical verbs, copulas and auxiliary verbs

The raw numbers of different kinds of verbs per utterance in 200 word samples for each group is shown in Tables 7–8. The results reveal that participants produced more lexical verbs in Kiswahili (M = 62.3) than in English (M = 46.7) for non-brain-damaged speakers. Agrammatic speakers also produced more lexical verbs in Kiswahili (Mean = 36.7) than in English (M = 33.5), albeit with a smaller margin in comparison to their non-brain-damaged counterparts.

Copulas in Kiswahili were marginally higher (M = 25.2) than English (M = 18.2) and so were auxiliaries: Kiswahili (M = 24.3); English (M = 18.5) for NBD participants. In comparison with non-brain-damaged speakers, the production of copulas and auxiliaries by agrammatic speakers was significantly reduced: copulas in English (M = 9.2) and Kiswahili (M = 7.2) and auxiliaries in English (M = 13) and Kiswahili (M = 6.2).

#### Verb argument structure

The argument structures of verbs produced in the two languages was analysed in a sample of 200 words for each subject. The analyses shown in percentages in Tables 9 and 10 reveal that non-brain-damaged participants produced more verbs without internal arguments in English (M = 63) and Kiswahili (M = 63.2). The verbs with one internal argument (transitive) were the second highest produced in both English (M = 45.3) and Kiswahili (M = 22.6). The analyses also show that there was very limited use of ditransitive verbs in both languages with percentage production of below 5%.

Results from agrammatic speakers with respect to argument structure were surprisingly the opposite of non-brain-damaged controls. They produced more verbs with one internal argument (transitive verbs; M = 50.4%) in English and Kiswahili (M = 55.6) than those without internal arguments (intransitive verbs; M = 23.9) in English and Kiswahili (31.7). However, verbs with two internal arguments (ditransitive verbs) were also hardly produced.

### General discussion

The variables selected in this study provided a basis to compare linguistic structures of the languages investigated. It was possible to ascertain differences between the spontaneous and narrative output of agrammatic speakers and that of non-brain-damaged participants. The analysis presented in this chapter describes and quantifies verb and argument production, and are therefore a good reflection of how agrammatism is manifested in bilingual speakers of English and Kiswahili. As mentioned earlier, with respect to spontaneous output in bilingual speakers of these languages, this is the first such attempt, hence opening up scope for more research. All participants had a first language (their mother tongue) prior to the acquisition of English and Kiswahili.

The results show a pattern of consistency in verb production between non-brain-damaged speakers and agrammatic speakers in both languages under investigation. The only exception is the agrammatic subject EA, who produced many errors and fell significantly below the normal range. Non-brain-damaged and agrammatic speakers produced longer utterances in English than in Kiswahili, although the difference was not significant. This could possibly be attributable to the differences in the configurations of the languages: Kiswahili is highly agglutinative, meaning that grammatical elements are attached to the verb, whilst English is more analytical. In Kiswahili, several morphemes are added to the verb to denote case, number, gender, person, and tense. Words are a combination of roots and stems, whilst in English, which is described as fairly analytic (Bickford, Albert & Daly, 1996) the vast majority of morphemes are free morphemes, that is, they are considered to be full-fledged ‘words’.

Another explanation is the fact that in Kiswahili, units that were counted as words are sentences when translated to English. For example, in English the three-word sentence ‘he beat him’ would be translated in Kiswahili as ‘*alimpiga*’. This was counted as a single word in this study and also as a one utterance. The unit of analysis was chosen as ‘utterance’ as described earlier, thus making comparisons between Kiswahili and English viable. Results of MLU analyses describe agrammatic output in English and Kiswahili. The findings were expected to be comparable to agrammatic spontaneous output research in Indo-European languages that showed short sentences are produced by agrammatic speakers of those languages (e.g. for English, Goodglass, 1976; Thompson, Shapiro, Li & Schendel, 1994; for Italian, Rossi & Bastiaanse, 2008). The same characteristics were found in the present study: shorter MLU in words and proportionately more simple sentences produced by agrammatic speakers.

As anticipated, the non-brain-damaged speakers did not omit or substitute verbs in obligatory contexts. However, verb production in agrammatic speakers showed a reduced number of lexical verbs, as reported by Thompson et al. (1994) for English, and Bastiaanse, Jonkers & Moltmaker-Osinga (1996) for Dutch. Auxiliaries were also reduced in agrammatic speakers in comparison with non-brain-damaged participants, supporting findings by Bastiaanse, Hugen, Kos & Van Zonneveld (2002) for Dutch and Miceli, Silveri, Romani & Caramazza (1983) for Italian.

The present data revealed that both groups produced more verb tokens in Kiswahili than in English, although the verb diversity was relatively higher in English. This means that Kiswahili spontaneous language produced by the participants had low lexical content in comparison to English. This could be explained by the use of compensation and adaptive strategies for Kiswahili due to the context of use. Whilst English is mainly used in formal situations in Kenya, Kiswahili is used in everyday conversation. So speakers tend to use verbs in obligatory conditions in English but not in Kiswahili, as long as they are understood by other interlocutors.

The production of copulas and auxiliaries in English and Kiswahili was found to be similar for non-brain-damaged participants in the two languages relative to the number of utterances. However, agrammatic speakers’ output was characterised by low levels of these linguistic units generally.

The analysis concerning verb argument structure surprisingly showed sharply dissimilar trends in production between the two groups of participants for both languages. Previous studies on argument production in spontaneous output, for example Thompson et al. (1997) who distinguished one (intransitive), two (transitive) and three (ditransitive) place verbs and counted the frequency of each, found that agrammatic speakers use relatively fewer two and three-place verbs than non-brain-damaged speakers. Rossi & Bastiaanse (2008), in their analysis of Italian agrammatic output, counted the number of internal arguments produced and found that agrammatic speakers produce significantly more verbs without internal arguments (one-place verbs) than agrammatic speakers and that there was no significant difference in the production of verbs with one or two internal arguments between the two groups. The results of the present study were comparable to those of Rossi & Bastiaanse (2008) with respect to non-brain-damaged speakers. The use of one-place verbs (intransitives) was higher than transitives for non-brain-damaged participants, whilst an opposite pattern was observed for their agrammatic counterparts who produced more transitive than intransitive verbs. The only similarity between the groups was found in the use of ditransitives, which was very minimal for both groups.

### Conclusion

This study reports findings of analyses conducted on the spontaneous and narrative output of English-Kiswahili bilingual agrammatic and age and education-matched non-brain-damaged speakers. It is the first effort as far as existing literature is concerned and hence provides novel data in verb and argument structure production in Kiswahili. The data elicited from picture description and narration in English and Kiswahili were compared for each group and between the groups. The results revealed a remarkable consistency among the participants in their verb production in both languages. The pattern of use of lexical verbs (token and types), copulas and auxiliaries was similar in the two languages for the participants. This suggests that they are well balanced bilinguals and hence suitable for this kind of cross-linguistic comparative study.

The overall finding in this study is consistent with results from similar studies in Indo-European languages. The output of agrammatic speakers is characterised by short, simple utterances with proportionately fewer grammatical morphemes. Inasmuch as the performances of most of the participants were comparable to those of non-brain-damaged controls, they still fell short in certain respects. This was especially observed in the total sample size recorded which averaged more than 500 words for non-brain-damaged participants and 200 for agrammatic speakers. This meant that for purposes of analysis (pegged at 200 words), entire samples were analysed from agrammatic speakers, whilst those from non-brain-damaged speakers were proportionally selected. Inflectional errors were found in samples from three agrammatic speakers, with EA particularly showing selective impairment. He had very high levels of omission of inflection morphemes in both languages.

The differences in variables found between the two languages were largely attributable to the contrast in the syntactic structures of the languages studied. English is described as being ‘fairly analytical’, whilst Kiswahili is classified as agglutinative (Bickford *et al*., 1996). The contrast was clearly shown by the significance in difference of the mean lengths of utterance produced by the participants in English and Kiswahili.

The study was found to be suitable for analysing verb and argument production in the spontaneous output of English-Kiswahili bilingual agrammatic speakers in Kenya. It provided insight on patterns of language storage in the brain with respect to participants who can be characterised as balanced bilinguals.

### Issues for future research

The agglutinative nature of the Kiswahili language as pointed out in the present study means that several affixes are glued together, essentially resulting in ‘single-word’ sentences. This study avoided the possible problems this structural aspect would have on the results by basing analyses on ‘the utterance’. However, it is the recommendation of this study that there is need for research to identify the demarcation of a word in Kiswahili as compared to English. The question of ‘what is a word?’ in Kiswahili is crucial in the comparison of verb production with other languages like English since this kind of studies use word samples in analyses.

The other challenge observed in Kiswahili-English spontaneous data was the propensity of participants to code-switch. We tried to avoid this pitfall by doing recordings for this study on different dates for the two languages, and even though this helped to a large extent, there were still quite a few code-switched data in the final transcripts. For analysis purposes for this study, these data were excluded and therefore did not have any effect on the results reported. However, future research can analyse the impact of code-switching in agrammatic spontaneous output, given that there are several studies on code-switching in output of non-brain-damaged individuals in the literature.

Finally, the Kiswahili narrative output revealed the use of what can be characterised as a narrative marker ‘*ka’.* Both non-brain-damaged and agrammatic individuals used this marker in telling stories depicted in the pictures. The past narrative marker (the -KA- tense) is used for narration, but it is often preceded in output by a first verb in the simple past. The past narrative exists only in the affirmative. The infix -KA- is placed between the affirmative subject prefix and the verb radical. This tense accommodates object infixes, but cannot be used in relative constructions. It was observed that participants used this marker frequently in narration and this could have had an impact on tense (time reference). This was beyond the scope of the present study and therefore we recommend studies focusing on the impact of this marker on time reference in Kiswahili narrative output.
